# Factors associated with mothers’ knowledge on infant and young child feeding recommendation in slum areas of Bahir Dar City, Ethiopia: cross sectional study

**DOI:** 10.1186/s13104-017-2510-3

**Published:** 2017-06-05

**Authors:** Yeshalem Mulugeta Demilew

**Affiliations:** School of Public Health, College of Medicine and Health Sciences, Bahir Dar University, P.O. Box 79, Bahir Dar, Ethiopia

**Keywords:** Mothers, Knowledge, Slum, Ethiopia

## Abstract

**Background:**

Malnutrition is a public health concern in Ethiopia. This might be correlated with inappropriate infant and young child feeding practice. This in turn is affected by Mothers’ knowledge on feeding practice. However, information on mothers’ knowledge on infant and young child feeding recommendation was scarce in Ethiopia. Therefore, this study was designed to assess mothers’ knowledge on infant and young child feeding recommendation and associated factors in slum areas of Bahir Dar City, Ethiopia.

**Methods:**

A community based cross-sectional study was conducted from May 1–26/2015. Systematic sampling technique was used to select respondents. Data were collected by pretested, structured, interviewer administered questionnaire. Data were entered and analyzed by SPSS version 20 software. Knowledge score was computed. Binary and multivariable logistic regression analysis were used to identify factors associated with maternal knowledge.

**Results:**

Only 28.7% of mothers had sufficient knowledge on infant and young child feeding recommendation. Factors associated with mothers, knowledge were above primary education [AOR 2.5, 95% CI (1.5, 3.9)], possession of radio [AOR 1.7, 95% CI (1.1, 2.7)], attending antenatal care [AOR 2.4, 95% CI (1.5, 4.0)], and having employed husband [AOR 2.3, 95% CI (1.2, 4.4)].

**Conclusion:**

Mothers’ knowledge on infant and young child feeding recommendation was very low. Hence, education on infant and young child feeding recommendation should be strengthened during antenatal care visit and using mass media especially for mothers with lower educational status to fill up of this gap.

## Background

In developing countries, malnutrition continues to be a major contributor to childhood morbidity and mortality. Thus, it adversely affects individuals, family and the community at large [1, 2]. Primarily, malnutrition occurs during the first 2 years of life as children have high demand for nutrients to support rapid growth and development; are more susceptible to infection; have heightened sensitivity to biological programming and are totally dependent on others for nutrition, care and social interactions [3]. Hence, the first 2 years of life is considered as a critical window of opportunity to address malnutrition [4].

From all proven preventive health and nutrition interventions infant and young child feeding (IYCF) has greatest potential impact on child survival [5]. Reduction of child morbidity and mortality can be reached only when IYCF practices are properly implemented to ensure adequate nutrition during early child hood period [6–8]. Despite strong evidence on benefit of IYCF, inappropriate IYCF practices have been widely documented in low- and middle-income countries [9–15].

Ethiopian government developed and implemented the Infant and Young Child Feeding (IYCF) guideline in 2004 to improve feeding practice [16]. Based on the guideline IYCF messages were given at health institution and community level. However, majority of mothers inappropriately fed their children [17–20]. Urban slum dwellers are illiterate with low socioeconomic status, and lack of access to basic health care facilities which further affect IYCF practice [21, 22].

Knowledge of the mother on IYCF recommendation has statistically significant association with their IYCF practice. Mothers who have insufficient knowledge on IYCF recommendation were more likely to have inappropriate IYCF practice [23, 24]. Even, inadequate knowledge is often an important determinant of care givers feeding practice than availability of food [6, 25]. Nevertheless, the rate of knowledge on IYCF recommendation differed across countries. Previous studies in various countries such as Zambia, Nigeria and Ghana have shown different level of mothers’ knowledge on IYCF recommendations [25–27].

Several factors affect mothers’ knowledge on IYCF recommendation. These factors include working status of the mother, family size, educational and marital status of the mother, age and sex of the child [2]. Even though, we have seen that different factors affect knowledge of the mother. Nonetheless, there are two rationales to conduct the study in slum sub-cities of Bahir Dar City. Firstly, the researcher hasn’t come across prior study in the area. Secondly, knowledge is affected by particular cultures of a given community, which make conducting a study in the target area appropriate. Therefore, this study was conducted to assess the level of mothers’ knowledge on IYCF recommendation and associated factors among mothers in slum areas of Bahir Dar City.

## Methods

The study was conducted in slum sub-cities of Bahir Dar City. The City is the capital City of Amhara Regional State, which is found at 565 km far from Addis Ababa, Northwest Ethiopia. The total population of the City is 288,200, of which 146,982 were females. According to the Bahir Dar City administration health bureau report, the numbers of under-5-year-old children and children aged 24–36 months were 4345 and 908, respectively. For administrative purpose the City is divided into nine sub-cities. Among which, three sub-cities are slum (Shumabo, Gish-Abay, and Sefene-selam). Majority of the residents in the slum sub-cities are daily laborers and petty traders.

In the City there are three hospitals (one public Regional referral hospital and one public and one private hospital), 6 health centers, 2 nongovernmental clinics, 9 private special higher clinic, 2 private higher clinics and 12 private clinics to deliver different health care services such as health promotion, prevention, curative and rehabilitative services to the community.

Community based cross-sectional study was conducted among mothers who have 24–36 months old children from May 1–26/2015. The sample size was calculated using Epi-info soft ware version 3.5.2 by considering the following assumptions: 95% confidence interval, 80% power, 52% proportion of knowledge on IYCF, odds ratio to be detected 2, Non-response rate 10%. The final sample size was 495.

Systematic sampling technique was used to select the study participants. The sample frame was list of mothers who have 24–36 months old children in the slum areas registered by the urban health extension workers. Using this registration logbook the study participants were selected by systematic sampling technique (every other child was selected) considering proportional to size allocation (by considering the number of 24–36 months children’s mother) for each slum area. In households with two women with 24–36 months old children, one was selected by lottery method.

Data were collected by pretested, structured interviewer administered questionnaire. The questions were adapted from different literature [6, 8, 28]. Interview with mothers was conducted at their home considering privacy. Three diploma nurses and one public health professional were recruited as a data collector and supervisor respectively.

Mothers’ knowledge on IYCF recommendation was assessed by twelve item questions which had yes or no response. A score of one was given for each correct response and zero for wrong response [29]. Mothers who had above six (those who score above average) were leveled as had “sufficient knowledge”, whereas, mothers who had six and below were leveled as had “insufficient knowledge” [24].

Data collectors and the supervisor were trained on the purpose of the study, data collection technique and tool before and after pretest. The questionnaire was pre-tested in slum area near Bahir Dar City and errors were corrected accordingly. Each day, collected information was reviewed and errors were returned to data collectors for correction. The supervisor and investigator closely supervised data collection technique.

Data were entered and analyzed using SPSS version 20. A frequency of each variable was calculated to check for accuracy, outliers, consistency and missed values. Mothers’ knowledge on IYCF recommendation was computed. Binary and multivariable logistic regression was done to identify factors associated with mothers’ knowledge. P value ≤0.2 was taken as a cut-off point to select variables for the multiple logistic regression models. Place of delivery, educational status, educational status of the husband, ANC follow up, occupational status, occupational status of the husband, religion, possession of television and radio were included in the final adjusted model. The model was built with backward elimination to avoid multicollinearity. A P value of less than 0.05 was considered statistical significant.

The study was approved by Ethical review Board of Bahir Dar University. Letter of permission was taken from Bahir Dar City administration health Bureau and sub-city administrators. Verbal consent was taken from participants. Privacy and confidentiality was maintained throughout the study period by excluding personal identifiers from the data collection form.

## Results

### Socio demographic characteristics

Among a total of 495 mothers sampled, 471 were participated, making the response rate of 95.1%. The mean age of the mothers was 30± (4.3 SD) years. Majority of the respondents were from Amhara ethnic group, 427 (90.7%) and orthodox Christian followers, 398 (84.5%). One hundred fifty-four (32.7%) mothers and their husbands, 101 (21.4%) had no formal education. Nearly half of the mothers, 225 (47.8%) and their husbands, 218 (46.3%) were housewives and daily laborers respectively (Table 1).

**Table 1:** Socio-demographic characteristics of mothers and their husbands in Slum areas of Bahir Dar City; Ethiopia, May 2015

Variable	Frequency (n = 471)	Percentage
Age of the mother (years)		
≤24	102	21.7
25–29	207	43.9
≥30	162	34.4
Mean ± (SD) age of their children = 29.65 ± (4.3)		
Religion		
Orthodox	398	84.5
Muslim	52	11.0
Protestant	21	4.5
Ethnicity		
Amhara	427	90.7
Others*	44	9.3
Educational status of the mother		
Have no formal education	154	32.7
Primary education	129	27.4
Secondary education and above	188	39.9
Occupational status of the mother		
Housewife	225	47.8
Daily laborer	147	31.2
Government employee	58	12.3
Petty trader/private employee	41	8.7
Marital status of the mother		
Married	404	85.8
Never married/widowed/divorced	67	14.2
Educational status of their husband		
Have no formal education	101	21.4
Primary education	110	23.4
Secondary education and above	260	55.2
Occupational status of their husband		
Daily laborer	218	46.3
Government employee	101	21.4
Carpenter/driver/private employee	152	32.3
Family size		
≤4	305	64.8
>4	166	35.2

Others* = Agew, Tigry and Oromo

### Mothers knowledge of IYCF

Overall, 135 (28.7%) mothers had sufficient knowledge on IYCF practice recommendation. Majority of the mothers knew the time of initiation of breast feeding, 432 (91.7%), the duration of exclusive breast feeding, 397 (84.3%) and when to switch to the other breast, 379 (80.5%). However, only few mothers were familiar with frequency of breast feeding per day, 142 (30.1) and total duration of breast feeding, 96 (20.4%) (Table 2).

**Table 2:** Knowledge of mothers regarding IYCF practice in Slum areas of Bahir Dar City, Ethiopia, May 2015 (n = 471)

Variable	Frequency	Percentage
A neonate should start breastfeeding within 1 h of birth		
Yes	432	91.7
No	39	8.3
An infant should exclusively breastfeed for the first 6 months		
Yes	397	84.3
No	74	15.7
An infant should breast feed at least 8 times/day (on demand but not <8 times)		
Yes	142	30.1
No	329	69.9
An infant should finish one breast milk before switch to other breast		
Yes	379	80.5
No	92	19.5
An infant should continue breastfeeding until 2 or more years		
Yes	96	20.4
No	375	79.6
An infant should start complementary foods at 6 months		
Yes	414	87.9
No	57	12.1
Abreast feed 6–8 months infant should take complementary food 2–3 times/day		
Yes	146	31
No	325	69
A breast feed 9–12 months infant should take complementary food 4 times/day		
Yes	84	17.8
No	387	82.2
A 6–23 month child need 4 or more food staffs		
Yes	64	13.6
No	407	86.4
Non breast feed infant needs extra meal		
Yes	314	66.7
No	157	33.3
An infant needs iron-rich foods		
Yes	87	18.5
No	384	81.5
Sick children need extra meal		
Yes	29	6.2
No	442	93.8
Over all knowledge of IYCF		
Sufficient knowledge	135	28.7
Insufficient knowledge	336	71.3
Source of information		
Health professionals	283	60.0
Mass media	134	28.5
Relatives	54	11.5

Four hundred fourteen (87.9%) respondents identified exact time of complementary feeding initiation. Even if, majority knew when to start complementary feeding, only a small number of mothers had knowledge on minimum number of complementary food staffs, 64 (13.6), frequency of feeding per day, 146 (31%) and need to increase feeding frequency when age increase, 84 (17.8%). Besides, only a small number of respondents were familiar about need of iron rich foods, 87 (18.5%) and extra meal during illness, 29 (6.2%) (Table 2).

### Factors associated with mothers’ knowledge

In the binary logistic regression analysis place of delivery, possession of television and radio, educational status of respondents and their husbands, occupational status of mothers and their husbands were statistically associated with mothers knowledge on IYCF recommendation (Table 3).

**Table 3:** Factors associated with mother’s knowledge regarding IYCF in Slum areas of Bahir Dar City; Ethiopia, May 2015

Factor	Mothers knowledge on IYCF		COR (95% c/I)	AOR (95% C/I)
	Sufficient	Insufficient		
Place of delivery				
Health institution	130 (27.6)	304 (64.5)	2.7 (1.0, 7.1)	
Home	5 (1.1)	32 (6.8)	1.00	
Possession of television				
Yes	103 (21.9)	192 (40.7)	2.4 (1.5, 3.7)	
No	32 (6.8)	144 (30.6)	1.00	
Possession of radio				
Yes	76 (16.2)	122 (25.9)	2.2 (1.5, 3.3)	1.7 (1.1, 2.7)
No	59 (12.5)	214 (45.4)	1.00	1.00
Educational status				
Less than secondary education	51 (10.8)	104 (22.1)	1.00	1.00
Secondary and above education	84 (17.8)	232 (49.3)	3.6 (2.4, 5.5)	2.5 (1.5, 3.9)
Educational status of the husband				
Less than secondary education	39 (8.3)	172 (36.5)	1.00	
Secondary and above education	96 (20.4)	164 (34.8)	2.5 (1.6, 3.9)	
ANC follow up				
Yes	105 (22.3)	190 (40.3)	2.6 (1.6, 4.2)	2.4 (1.5, 4.0)
No	30 (6.4)	146 (31.0)	1.00	1.00
Occupational status of the mother				
Daily laborer	25 (5.3)	122 (25.9)	1.00	
Housewife	64 (13.6)	161 (34.2)	1.9 (1.1, 3.2)	
Petty traders and government/private employee	46 (9.8)	53 (11.2)	4.2 (2.3, 7.5)	
Occupational status of the husband				
Daily laborer	27 (5.7)	153 (32.5)	1.00	1.00
Government employee	38 (8.1)	63 (13.4)	3.3 (1.9, 5.4)	2.3 (1.2, 4.4)
Carpenter/driver/private employee	70 (14.8)	120 (25.5)	3.4 (1.9, 6.0)	2.1 (1.2, 3.6)
Religion				
Orthodox	122 (25.9)	13 (2.8)	2.0 (1.0, 3.8)	
Muslim and protestant	276 (58.6)	60 (12.7)		

*AOR* adjusted odds ratio, *COR* crude odds ratio, *95% CI* ninety-five percent confidence interval

In the multivariable logistic regression analysis, having beyond primary education [AOR 2.5, 95% CI (1.5, 3.9)], possession of radio [AOR 1.7, 95% CI (1.1, 2.7)], attending antenatal care [AOR 2.4, 95% CI (1.5, 4.0)], and having employed husband [AOR 2.3, 95% CI (1.2, 4.4)], were positively associated with mothers knowledge on IYCF recommendation (Table 3).

## Discussion

Mothers who have sufficient knowledge on IYCF recommendation were more likely to have better feeding practice than mothers who have insufficient knowledge [24, 30, 31]. However, in this study only 28.7% of mothers had sufficient knowledge on IYCF recommendation. Inappropriate knowledge on IYCF recommendation was also reported from previous study finding in Hebei province, North China [29].

On the other hand, this finding was lower than the study findings in Shashemene Woreda, Mekelle, Dehradun District and Nigeria [20, 26, 32, 33]. This discrepancy might be due to time gap between studies and difference in the study settings; since the former studies included mothers in the Woreda with better socio economic characteristics whereas the current study was done among mothers in slum area with lower socio economic status.

Early initiation of breastfeeding plays a crucial role for a significant reduction of neonatal and infant mortality [34, 35]. Besides, it has benefit to the health of the mother as early suckling stimulates the release of prolactin, which helps in the production of milk, and oxytocin, which is responsible for the ejection of milk and stimulates the contraction of the uterus after childbirth [6]. Moreover, initiation of breast feeds within 1 h of birth is the key for successful breast feeding [36]. Mothers’ knowledge on initiation of breast feeding is an important determinant factor for their practice. In the present study majority of the mothers (91.7%) knew exact time when to initiate breast feeding after delivery. This finding was better than the study findings in Mizan Aman town, Ethiopia, Nigeria and Nepal [37–39].

Exclusive breast feeding for the first 6 months of life has a significant effect in the reduction of morbidity and mortality as well as to ensure overall development [40]. Despite the well-recognized importance, exclusive breastfeeding practice is not widespread in the developing countries. In the present study, 84.3% of mothers had knowledge on the need of exclusive breastfeeding for the first 6 months. This finding was better than the study findings in Mizan Aman town, Ethiopia and Andhra Pradesh, India [37, 41]. This might be due to time gap; currently there is nutrition education on IYCF practice through mass media and professionals which has direct contribution to the improvement of maternal knowledge [42].

Continuation of breast feeding up to the age of 2 years or beyond is important as it provides useful amounts of energy, good quality protein and other nutrients essential for brain development [8]. However, in the present study, only 20.4% of mothers knew that breast feeding is to be continued for 2 years or beyond.

In Ethiopia, 10% of infants under-6 months are given complementary foods in addition to breast milk and 49% of children age 6–8 months consume solid, semi-solid, or soft foods. Among breastfed children age 6–23 months, only 4% receive foods from at least four foods groups (the minimum acceptable diet), while 48% are fed the minimum number of times or more [2]. In the present study, 87.9% of mothers knew correct age of introduction of complementary feeds. Knowledge of mothers on frequency and quantity of complementary feeds was very low (17.8 and 13.6% respectively). This finding was consistent with study finding in Ghana [43]. This low level of knowledge might be due to low attention of participants during nutrition education, often diversity and frequency of complementary food is discussed near the end of education section.

Educational status of the mother had statistically significant association with mothers’ knowledge on IYCF practice. Mothers who attained beyond primary education were 2.5 times more likely to have sufficient knowledge than mothers who attained lower than secondary education. Similar finding was reported from previous studies [32, 44–46]. This might be due to the fact that literate mothers understand nutrition information given by the health and nutrition programs.

Having radio was statistically significant factor to improve mothers’ knowledge. Mothers who have radio were 1.7 times more likely to have sufficient knowledge than their counter part have no radio. Similar result was reported from a study finding in Arba Minch Zuria [47]. Respondents who have radio were more likely to get nutrition related information from nutrition education given by radio [26].

In the study area each woman who comes to the health institution to get immunization service, to attained antenatal and postnatal care is counseled on maternal and child nutrition. In addition to this, in each health facility, theoretical and practical education on IYCF practice is given one times per month during “mothers’ day”. In this “mothers’ day” complementary feeding preparation is demonstrated by health professionals. Moreover, education on IYCF practice is given in the health post to the women who come to get immunization service, to attained antenatal and postnatal care by health extension workers (HEWs). Furthermore, HEWs give nutrition education to all pregnant and lactating women at their home. All these have direct contribution on maternal knowledge. However, according to Ethiopia Mini Demographic and Health Survey done in 2014, 58% of pregnant women who gave birth in the 5 years preceding the survey received antenatal care for their most recent birth, 34% from a nurse or midwife, 6% from a doctor and 18% of women from a health extension worker. Antenatal care utilization was defined at least one visit of health institution for checkup purpose during the pregnancy of the index child [48].

In this study mothers who had ANC follow up were 2.4 times more knowledgeable than their counter parts who had no ANC follow up. This finding is in agreement with the study finding in northern Ethiopia and Arba Minch Zuria [47, 49]. Mothers who had ANC follow up were more likely to be counseled by professionals on IYCF, which have direct contribution to improve their knowledge level [50]. The fact that this study did not use qualitative information on the guideline to probe their knowledge level and the study includes mothers who have children 24–36 months old are the limitation of this study.

## Conclusion and recommendation

Knowledge of mothers on IYCF practice especially on frequency and type of complementary food, need of iron rich foods and additional foods during illness was very low. Hence, education on IYCF practice should be strengthen by giving emphasis to frequency and type of complementary food, need of iron reach foods, need of additional food during illness and importance of continuing breast feeding up to 2 years by health professionals and through radio can help in filling up of this gap. Improvements should be made in social conditions including literacy, use of radio and major social mobilization endeavors to improve ANC utilization.

## Abbreviations

ANC: Antenatal Care
IYCF: Infant and Young Child Feeding
PNC: Postnatal Care
